# The effect of earthquakes on the housing market and the quality of life in the province of Groningen, the Netherlands

**DOI:** 10.1007/s10901-018-9600-y

**Published:** 2018-02-22

**Authors:** Peter Boelhouwer, Harry van der Heijden

**Affiliations:** OTB Research for the Built Environment, Faculty of Architecture and the Built Environment, Delft University of Technology, P.O. Box 5043, 2600 GA Delft, Netherlands

**Keywords:** Earthquakes, Housing market, Province of Groningen, Effects of gas extraction, House prices

## Abstract

The Netherlands is not known for the occurrence of earthquakes. This is, however, a hot topic in the province of Groningen. Because of gas extraction, this area suffered from more than 1000 earthquakes. Most of them are not very intensive, but also bigger earthquakes of between 2 and 3 on the scale of Richter have been measured. In the last few years, thousands of houses are damaged, house prices have dropped, and the liveability is at stake. This paper pays attention to the effects of these earthquakes on the functioning of the housing market and the quality of life in the region. An extensive survey was conducted, focus groups were organized, housing market statistics were analyzed, and several econometric models were evaluated to give an answer to the question in which way the earthquakes influence the local housing market and the liveability.

## Introduction

**Fig. 1:**
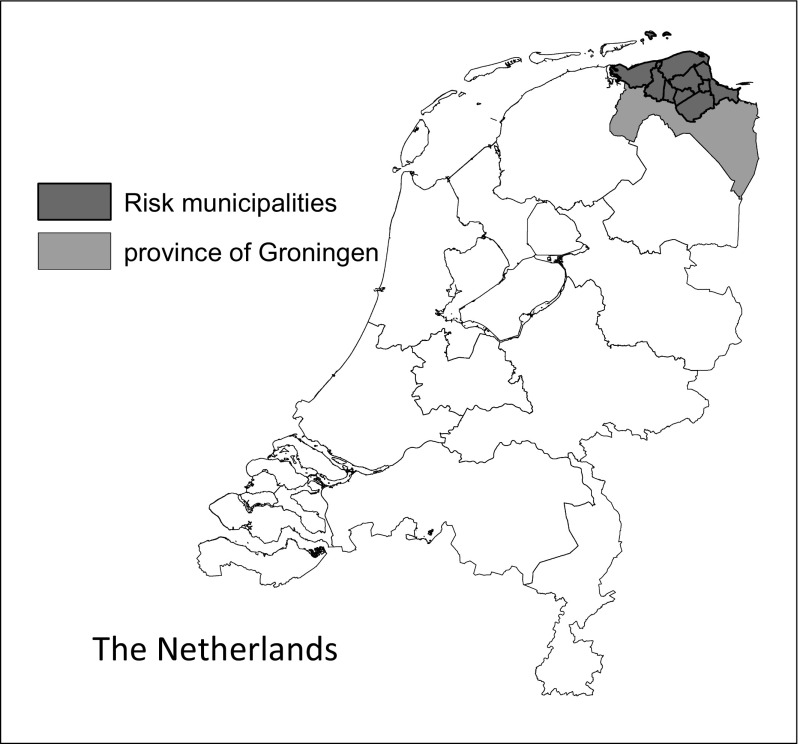
Risk municipalities in the province of Groningen in the Netherlands

Since 1963, natural gas has been extracted in the Dutch Province of Groningen. This province is located in the north of the Netherlands and is adjacent to Germany (Fig. 1). The natural gas yield for the Dutch state is now amounting to about 250 billion euros. The bulk of it is generated by the Groningen gas field (Commissie Duurzame Toekomst Noord-Oost Groningen 2013). The natural gas is mined by the Dutch Petroleum Company (NAM), a joint venture of Shell and ExxonMobil. NAM expects to be able to produce natural gas in Groningen for another 50 years.

The extraction of natural gas in Groningen has benefits such as the creation of new jobs, financial revenues for the state, but has also disadvantages such as soil subsidence and earthquakes since 1986. The KNMI (Royal Netherlands Meteorological Institute) has already registered more than 1000 earthquakes in the northern part of the Netherlands. Most of these are rather light and not stronger than 2.0 on the Richter scale (KNMI 2016). But heavier quakes have occurred, about 100 with a magnitude between 2.0 and 3.0 and 14 with a force greater than 3.0 (Rijksoverheid 2016). The strongest earthquake occurred on August 2012 at Huizinge (municipality Loppersum) and had a magnitude of 3.6 on the Richter scale.

A magnitude of 3.6 does not seem very much, but the Richter scale is primarily used for tectonic earthquakes which occur very deep. Because the Groningen gas field is only a few kilometers below the ground, these relatively light earthquakes have caused damage to buildings. By the end of 2016, approximately 75,000 claims for damaged buildings were submitted to the NAM. But the effects go further and also affect the quality of life of households in the area. Ultimately, it also influenced the housing market, especially after the relatively strong earthquake at Huizinge in August 2012. The consequences of the earthquakes for the housing market are crossed by a crisis on the Dutch housing market from 2009. The lowest point of this crisis was reached in 2013.

In 2015, a comprehensive study of the effects of earthquakes on the housing market in the province of Groningen in the Netherlands was conducted (Boelhouwer et al. 2016), which paid particular attention to:

- The living experience and quality of life;
- The housing choices of households;
- The house price development and marketability of dwellings.

These aspects were elaborated in several monographs (Boelhouwer et al. 2016; Boumeester 2016a, b; Hoekstra 2016; van der Heijden 2015; Jansen et al. 2016; Simon et al. 2016). The research focused on the nine municipalities that were most affected by the earthquakes: Appingedam, Bedum, Delfzijl, the Marne, Eemsmond, Loppersum, Slochteren, Ten Boer and Winsum.

A survey among inhabitants was organized to establish the effects of the earthquakes on the living experience and quality of life of the local residents of the nine risk municipalities. Additional to this quantitative survey, a residents survey in the form of 11 group meetings and 4 larger residents’ meetings was organized (Simon et al. 2016).

The impact of the earthquakes on the actual choice behavior of households was examined through migration research (Boumeester 2016a) and an analysis of developments in the housing market (van der Heijden 2015). In addition, an indicator that shows the development of confidence of households in the housing market was developed (Boumeester 2016b). Finally, using the results of the residents survey on willingness to move, the possible future choice behavior of households in the risk municipalities was established (Hoekstra 2016). Because several scholars already developed statistical models for mapping the effects of earthquakes on the value of owner-occupied housing, no specific quantitative model was developed, but a counter appraisal was carried out on existing models to test the usefulness of these models (Jansen et al. 2016).

In this paper, a general overview of the main results from all the underlying studies is presented according to the three aspects mentioned above: living experience and quality of life, residential mobility, housing market and house price developments (see Boelhouwer et al. 2016). These results are the basis for the policy recommendations in the last section. Prior to the presentation of these results, Sect. 2 addresses the way in which the investigation within the nine municipalities was organized. In Sect. 3, the quality of life and the living experiences are under study. The preference to move and housing market developments, including house price developments, are elaborated in Sect. 4. This contribution ends with some conclusions and policy recommendations.

## Zoning of the risk area

The risk area for earthquakes is broken down by the share of dwellings with earthquake damage (damage intensity) and by demographic developments.

### Damage intensity

As explained, the housing market research focuses on nine risk municipalities in the province of Groningen. It’s important to realize that the impact of the earthquakes differs quite a lot within and between these municipalities. And thus, the impact of the earthquakes differs in the housing market, from place to place. It is therefore important to make a distinction between different sub-areas within the risk area. Municipalities are not a good basis for this, because as explained the intensity and frequency of earthquakes within municipal boundaries are not similar.

**Fig. 2:**
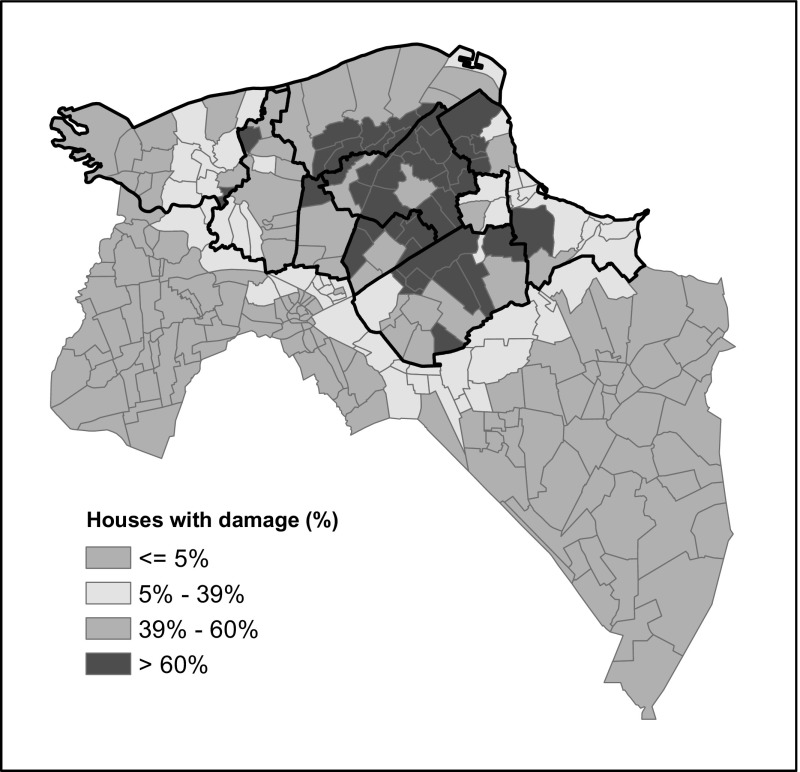
Percentage of houses with damage in four classes per zip code area. The nine risk municipalities are marked with a black line. *Source*: Boelhouwer et al. (2016, p. 25)

In a study of Koster and Van Ommeren (2015), the price development of houses as caused by earthquakes is studied on the basis of the severity and frequency of earthquakes in different locations. However, the question is whether this is the best approach to study differences in the effect on the housing market (c.q. differences in the price development) that earthquakes have in various locations. The consequences of earthquakes for the housing market in particular will be determined by the extent to which dwellings have been damaged. That depends not only on the intensity and frequency of the earthquakes, but also on the soil and characteristics of the dwellings, such as the year and type of construction. Therefore, it was decided to use the damage to dwellings caused by earthquakes as a basis for the zoning. On the basis of NAM data, available on accepted claims per zip code area in the province of Groningen, we first established the proportion of dwellings with earthquake damage. On the basis of this information, the next step was to divide the area into four ‘damage intensity’ classes (Fig. 2). During the research, the two classes with the lowest share of damaged dwellings have been merged.

### Population growth

In addition to the earthquakes, the shrinking population plays a major role on the housing market in parts of Groningen. More specific, the decrease in the number of households is central in this development (VNG 2009). A declining population often goes hand in hand with declining birth rates and an aging population. This process puts the socioeconomic base of a region under pressure and makes it more and more difficult to maintain facilities such as shops, clubs and schools. Eventually, the quality of life is compromised. A shrinking population also has implications for the housing market: The demand for housing decreases.

To be able to ‘correct’ for the effects of a shrinking population on the housing market, the risk area is further divided into a part with a shrinking population (five municipalities) and a part where the population is not shrinking (four municipalities).

## Quality of life and living experience

One of the most important conclusions from the survey and the focus groups among the residents is that the quality of life in the earthquake area is seriously affected by the earthquakes. Thus, the quality of life in the Groningen earthquake area deteriorated significantly since the relatively strong earthquake of Huizinge in August 2012. In 2012, satisfaction with the neighborhood in the earthquake zone was more or less similar to the rest of the Netherlands; 85 and 86% of the residents were (very) satisfied (Hoekstra 2016). In 2015, satisfaction with the neighborhood, however, fell sharply in the earthquake area: Only 77% of the population was (very) satisfied. This still seems to be a reasonable score, but it belongs to the lowest scores in the Netherlands. On average, 85% of the Dutch population was (very) satisfied with their neighborhood in 2015 (MBZK 2016). The risk of this fall in satisfaction is that in the future more people will leave the area, and respectively, fewer new people will enter. This can lead to rising vacancy rates and fewer services and businesses which also reduce employment and start a vicious circle in which the area is becoming less attractive to settlers. In addition, many residents are afraid of (a further) damage to the cultural and landscape heritage in the region. The impact of earthquakes on the quality of life is a part of a gradual process on the long term. However, in the short and medium term, the earthquakes also have several negative effects on the well-being and housing satisfaction:

- Feelings of insecurity and other psychosocial and health problems caused by the earthquakes;
- Concerns about the performance and marketability of the property;
- Difficult and time-consuming procedures for compensation and handling the claims for repair;
- A growing distrust in national politics and a feeling that the problems of the people from Groningen are not taken seriously.

Of the nearly 53,000 households in the nine earthquake municipalities, over 15,000 households feel unsafe (Table 1), that is, 29% of the total number of households. Also, many people no longer feel safe in their own homes. This creates mental problems in nearly 4000 households (Hoekstra 2016).

**Table 1:** Response on the thesis: ‘I feel safe in my house’ by degree of earthquake intensity

	Completely agree (%)	Agree (%)	Neutral (%)	Not agree (%)	Fully not agree (%)	Do not know (%)	Population (weighted)	Respondents (not weighted)
Lower	10	44	29	11	3	2	20,026	1089
Average	10	43	26	16	5	1	27,043	2090
High	9	37	27	21	6	1	7117	1065
Total	10	42	27	15	4	1	54,186	4244

*Source*: Hoekstra (2016, p. 60)

The concerns about the price development and the marketability of the houses are connected to both the occurrence of earthquakes and population decline. Many homeowners are wondering whether they should still invest in their homes now that the value of their houses drops. The general feeling is that people cannot leave because their house is unsaleable. Half of the homeowners who are considering moving are wondering whether they are able to sell their property within 2 years (Hoekstra 2016). The handling of the claims for compensation and the connected procedures give residents with damaged houses in the earthquake area much concern and frustration (Simon et al. 2016). The residents have the opinion that there are no clear criteria for the determination and the settlement of the damage. Contradictory reports from experts are fueling the doubts the residents have on their expertise, objectivity and independency. Because of the slow and lingering procedures, some people feel that the earthquake problems and everything connected with it control their life completely. The residents are also concerned about the fact that the NAM seems to have eyes only for visible (cosmetic) damage above the ground and not for the damage to foundations and the underground infrastructure. They indicate that the assessment of the injury of their houses must be drawn wider than the damage by earthquakes only. Damage due to soil subsidence and other effects of the gas extraction must also be honored. Actually, the debate should be organized on the issue of gas extraction and not on earthquake issues only.

The residents seriously believe that the government and the NAM are doing too little to tackle the negative effects of the earthquakes (Simon et al. 2016). Many people have little or no confidence in the government any more. They do not feel heard and not taken seriously. The feeling prevails that the government takes the side of the NAM and does not take full responsibility for the resulting problems. The residents do not feel treated fairly.

## Residential mobility, housing market and house price developments

### Preferences to move

**Fig. 3:**
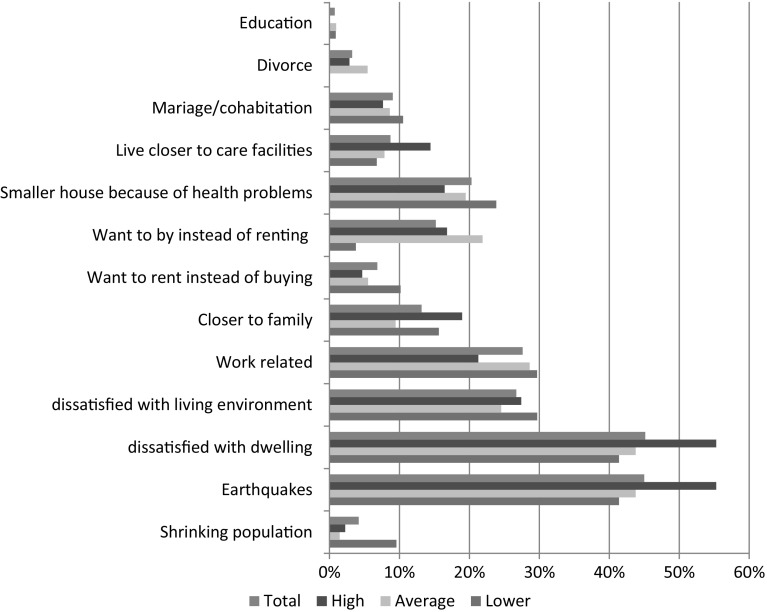
Reasons to move of households who want to move within 2 years to earthquake intensity (possibility of three choices). *Source*: Hoekstra et al. (2016, p. 39)

For some of the residents, the negative effects of the earthquakes are the main reason for the wish to move, and in many cases to leave the earthquake area. For 45% of the households who definitely want to move within 2 years (the effects of), the earthquake is one of three main reasons for moving (Fig. 3).

The proportion of households that definitely or maybe want to move within 2 years is in 2015 in the earthquake area by 10 and 28% significantly higher than in 2012 (7 and 16%). Compared to national figures, in the earthquake area willingness to move was lower in 2012 but is higher in 2015 (Table 2). It is obvious that the earthquake-related problems, which after 2012 (Huizinge) have become quite manifest, play an important role.

**Table 2:** Willingness to move in the earthquake area compared to the Netherlands, 2012 and 2015

	Definitely move within 2 years (%)	Maybe move within 2 years (%)
Earthquake area 2012^b^	7	16
the Netherlands 2012^b^	10	19
Earthquake area 2015^a^	10	28
the Netherlands 2015^c^	9	25

*Source*: ^a^Hoekstra (2016, p. 38), ^b^WoON 2012, ^c^WoON 2015 (national housing survey with more than 60,000 respondents)

Particularly, the proportion of households that might move (28%) has increased since 2012 in the earthquake zone. This is probably because earthquakes create uncertainty about the future housing situation and future living quality. It also appears that many more residents in the earthquake zone, despite a strong bond with the region, want to leave their current municipality than in a ‘normal’ housing market is common. The fact that especially the younger and more educated households want to move relatively more can have an additional negative effect on the quality of life in the area. Not only the earthquakes, but also the shrinking population plays an important role. In municipalities with a shrinking population and in municipalities with a high proportion of homes with earthquake damage, households are inclined to move more and are less satisfied with the neighborhood than in municipalities with a growing population and in areas with a relatively small proportion of homes with earthquake damage (Hoekstra 2016). Important for future policies is that also households who might move indicate that moving or not depends to a large extent on government policies and the situation on the housing market. Government interventions such as a purchase scheme, a fair compensation for the loss of value, the realization of earthquake-resistant housing and measures to increase the quality of life will largely determine which part of the moving-inclined residents will move eventually.

No less than 51% of homeowners inclined to move want to leave the province, compared to 26% of the tenants (Hoekstra 2016). So the earthquake problem has a greater impact on the home-ownership housing market than on the rental market. If the wishes of those who want to move are realized, it is likely that there will occur a large surplus of especially single-family dwellings in the earthquake area. The fact that especially the young and educated households want to move relatively frequently can have an additional negative effect on the quality of life in the area.

### Housing market developments

When we take a closer look at the housing market developments in the earthquake area, it appears that there are three partially additive effects. First, there is of course the general downturn in the housing market that manifested itself in the Netherlands as a result of the banking crisis in the third quarter of 2008. In addition, two other aspects have an important effect on the Groningen housing market: the earthquake problems, especially in the period after the earthquake in Huizinge in August 2012, and the shrinking population in parts of the area. Particularly, these last two aspects form a serious problem for the future. The combination of the crisis, the shrinking population and the earthquakes leads to a dysfunctional housing market in the province of Groningen. In the Netherlands, this was actually also the case in 2013 where at each property sold, there were 30 homes for sale. In the earthquake zone, this market indicator was as high as 54, almost double the average of the national figure. A housing market that is facing such an extreme supply/demand ratio can no longer be seen as a normal market with stable pricing.

From the second half of 2013, the Dutch housing market shows significant signs of recovery. For the Netherlands, the market indicator decreased in 2015 to the value of 11. However, it still remains a buyer’s market. Only in metropolitan housing markets such as Amsterdam and Utrecht, the market has turned into a sellers’ market and further price hikes are expected. This is contrary to the earthquake area where the market indicator, although decreased, still comes out at a very high level of 24 in 2015.

The housing market in Groningen is clearly influenced by the shrinking population in parts of the province, but both inside and outside the shrinking areas the housing market in risk municipalities appears to be lagging behind the recovery of the housing market in non-risk municipalities. This is not only reflected by the development of the market indicator, but also by the development of the number of dwellings sold, the difference between the transaction price and asking price, the number of homes for sale and the average time it takes to sell a house (van der Heijden 2015). This is reflected in the development of trust in the housing market as measured by the property market indicator as established monthly by the umbrella organization of owner-occupiers in the Netherlands (Vereniging Eigen Huis). The trust households have in the housing market rises in the fourth quarter of 2015 in the risk municipalities to 106, while the other municipalities in Groningen arrive at a score of 110 (Boumeester 2016b).

With regard to house price developments, it was concluded that, depending on the manner in which the risk area is defined and the reference areas are selected, the house prices in the earthquake area are on average lagging approximately 2% behind the prices in the reference areas (Jansen et al. 2016). Partly because of the limited number of sales, the accuracy of the model results is small (a large spread around the values) and sometimes not even statistically significant. However, it is demonstrated by several studies that the impact of the earthquakes on price developments is influenced by the number of damaged dwellings in the immediate vicinity (Jansen et al. 2016). As a result, behind the average price development in the earthquake region there may be large differences in the development of house prices between different areas within the region. To provide more insight into this effect, further research is necessary on a smaller geographical scale. But this is only possible when the number of transactions increases.

## Conclusions and policy recommendations

In this article, the main findings of a comprehensive study of the effects of earthquakes on the functioning of the housing market and the liveability in the nine municipalities in the earthquake area in the province of Groningen were presented. The most striking results are the sense of uncertainty and insecurity, the reduced quality of life, the lack of trust and transparency with regard to the policies and procedures by the residents in the earthquake area.

In general, the greater the earthquake intensity is (measured on the basis of the damage intensity), the greater the impact of the earthquakes is on the housing market. But also the inhabitants of areas with less earthquakes experience clearly the negative impact of the earthquake problem. In short, everywhere in the earthquake zone, earthquakes have a substantial impact on liveability and the quality of life whereby the earthquake intensity (along with a number of other determinants such as population decline) then provides a detailed spatial differentiation of the impact. This result underlines the effect of reputation. For many people, especially from outside the region, the whole area of the northeast of the province of Groningen is heavily related with earthquakes and no differentiation is made between areas with more and less damage. Subjective perceptions partly influenced by huge national media coverage play an important role in the effects of the earthquakes on the housing market and the liveability. Another finding is that until now, the average decrease in house prices as a result of the earthquakes of approximately 2% (based on dwellings that were actually sold) appears to be less of a problem than the marketability of dwellings and the drop in the amount of housing transactions. However, there may be large differences in the development of house prices between different areas within the region.

To be able to get a clearer picture of house price developments within the earthquake area and to be able to evaluate the effects of future policies, it is important that the development of the housing market in the earthquake area is monitored well.

On the basis of the results from the research, it can be concluded that future policies should be primarily focused on the removal of the adverse effects of the earthquakes: strengthening buildings, improving the handling and repair of damaged properties and offering a transparent and generous compensation for the fall in the value of properties. ‘Taking care’ of residents by establishing a focal point for citizens can play an important role in retaining trust of the residents in the government. The importance of such measures is demonstrated, for example, by the fact that a substantial part of the residents who are inclined to move out of the earthquake area indicate that they will probably stay if the gas drilling will be dramatically reduced, the earthquake damage to their property will be quickly repaired or if their current home will be made earthquake resistant.

In addition, it is very important that the psychosocial and health problems of residents as a result of the earthquakes get serious attention, that the level of facilities like schools, shops and healthcare facilities is improved and that residents and organizations in the area will be involved in the formulation and implementation of policies.

## References

[CR1] Boelhouwer P, Boumeester H, Grisnich F, Groetelaers D, de Haan F, van der Heijden H, Hoekstra J, Jansen S, Korthals Altes W, Ringersma R, Simon C, de Wolff H (2016). Woningmarkt-en leefbaarheidsonderzoek aardbevingsgebied Groningen.

[CR2] Boumeester H (2016). Migratiestromen in Noord-Oost Groningen.

[CR3] Boumeester H (2016). Eigen Huis Marktindicator-regionaal.

[CR4] Commissie Duurzame Toekomst Noord-Oost Groningen. (2013). *Vertrouwen in een duurzame toekomst; een stevig perspectief voor Noord*-*Oost Groningen*, Groningen (provincie Groningen).

[CR5] Hoekstra J (2016). Wonen en aardbevingen in Groningen. Een onderzoek in negen gemeenten.

[CR6] Jansen S, Boelhouwer P, Boumeester H, Coolen H, de Haan J, Lamain C (2016). Beoordeling woningmarktmodellen aardbe-vingsgebied Groningen.

[CR7] KNMI (Royal Netherlands Meteorological Institute). https://www.knmi.nl/kennis-en-datacentrum/uitleg/aardbevingen-door-gaswinning. Consulted 3 June 2016.

[CR8] Koster, H. R. A., & van Ommeren, J. (2015). *Natural gas extraction, earthquakes and house prices*. Tinbergen Institute discussion paper TI 2015-038/VIII.

[CR9] MBZK (Ministry of Internal Affairs) (2016). Wonen in Beweging. Resultaten van het WoonOnderzoek Nederland 2015.

[CR10] Rijksoverheid (National Government). https://www.rijksoverheid.nl/onderwerpen/aardbevingen-in-groningen/inhoud/aardbevingen-door-gaswinning-in-groningen. Consulted 3 June 2016.

[CR11] Simon C, De Haan F, Grisnich F, Ringersma R (2016). Wonen en leven met aardbevingen. Meningen, knelpunten en oplossingsrichtingen van burgers.

[CR12] van der Heijden H (2015). Ontwikkelingen op de markt van koopwoningen in Groningen.

[CR13] VNG (Association of Dutch Municipalities) (2009). Krimpen met kwaliteit.

